# Chronic Cranial Windows for Long Term Multimodal Neurovascular Imaging in Mice

**DOI:** 10.3389/fphys.2020.612678

**Published:** 2021-01-22

**Authors:** Kıvılcım Kılıç, Michèle Desjardins, Jianbo Tang, Martin Thunemann, Smrithi Sunil, Şefik Evren Erdener, Dmitry D. Postnov, David A. Boas, Anna Devor

**Affiliations:** ^1^Biomedical Engineering, Boston University, Boston, MA, United States; ^2^Centre de recherche du CHU de Québec, Université Laval, Quebec City, QC, Canada; ^3^Department of Biomedical Engineering, SUSTech, Shenzhen, China; ^4^Institute of Neurological Sciences and Psychiatry, Hacettepe Üniversitesi, Ankara, Turkey; ^5^Department of Biomedical Sciences, University of Copenhagen, Copenhagen, Denmark

**Keywords:** imaging, multimodal, vascular, neural, mice, awake, chronic

## Abstract

Chronic cranial windows allow for longitudinal brain imaging experiments in awake, behaving mice. Different imaging technologies have their unique advantages and combining multiple imaging modalities offers measurements of a wide spectrum of neuronal, glial, vascular, and metabolic parameters needed for comprehensive investigation of physiological and pathophysiological mechanisms. Here, we detail a suite of surgical techniques for installation of different cranial windows targeted for specific imaging technologies and their combination. Following these techniques and practices will yield higher experimental success and reproducibility of results.

## Introduction

The utilization of multiple imaging modalities in neuroscience enables comprehensive investigation of brain structure and function. Each imaging technology has unique advantages and disadvantages. For example, while ultrasound (US) allows whole-brain imaging of cerebral blood flow, it cannot achieve micron resolution. On the other hand, two-photon (2-P) microscopy yields micron resolution but is limited by relatively shallow penetration (in common practice, limited to the upper cortical layers) and relatively small field of view (usually, <1 mm).

Previous studies have described design and surgical implantation of chronic cranial windows in mice enabling longitudinal measurements (Mostany and Portera-Cailliau, 2008; Holtmaat et al., 2009; Andermann et al., 2010; Goldey et al., 2014; Roome and Kuhn, 2014; Heo et al., 2016). These window implants allow imaging of awake mice, which is important for investigation of brain function without the confounding effects of anesthesia on the physiology of nervous and cardiovascular systems. Although some of these protocols are highly detailed, they usually employ imaging with one system. Here, we expand on these previous publications and describe a spectrum of surgical methods in mice suited for multiple imaging modalities, used alone or in combination, including 2-P microscopy, laser speckle contrast imaging (LSCI), intrinsic optical signal imaging (IOSI), optical coherence tomography (OCT), and functional US (fUS). These optical windows can last for a span of up to 6 months after surgery and can be made MRI-safe for combined optical imaging or optogenetic (OG) stimulation in awake mice undergoing fMRI.

## Methods

The protocols described below contain surgical procedures as well as pre- and post-operative measures. Common procedures are presented in the main text. Procedures specific for individual imaging modalities are presented in the Supplementary Material. All surgical procedures and imaging protocols were approved by the institutional Animal Care and Use Committee.

### Pre-operative Measures

Adequate planning and preparation decrease the time spent during surgery and the risk of infection or inflammation leading to an overall increase in the success rate of surgery.

#### 4 A's: Anesthesia, Analgesia, Antibiotics, and Anti-inflammatories

In our procedures we have used isoflurane for anesthesia, buprenorphine for analgesia, ibuprofen for analgesic, and anti-inflammatory properties, dexamethasone for prevention of inflammation and brain edema (Hedley-Whyte and Hsu, 1986) and cefazolin and trimethioprim-sulfametoxazole as broad-spectrum antibiotics. For details, please see the Supplementary Material.

#### Sterilization and Preparation of the Surgical Room

The surgery room should be prepared before the start of the surgery. Surfaces should be cleaned with antiseptic solution and clutter should be avoided. There should not be any non-essential personnel traffic into the room. Surgical chair should be comfortable and adjustable. Surgeon should don personal protective equipment including lab coat, surgical mask, and gloves.

All tools and supplies used for surgery, starting from dissection of the skin, need to be sterile. Surgical tools and metal head bars should be autoclaved. If a surgical tool tip touches a non-sterile surface during the surgery, it should be bead sterilized. A metal tool tray should be autoclaved. Batches of paper cleaning wipes (e.g., Kimwipes) and glass pipettes can be autoclaved. Saline can be autoclaved in glass bottles (~10 ml) or purchased in sterile 10-mL batches. Gelatin absorbable hemostatic sponge (e.g., Surgifoam), cotton-tipped applicators, and bone wax are purchased in sterile batches. Thirty minutes before surgery, heating blanket, and hot bead sterilizer are switched on. The surgical microscope is adjusted (height and focus are in the middle of their dynamic range). Surfaces, trays, and handles are cleaned with an antiseptic solution.

Although every surgeon may feel more comfortable with a different set of tools and supplies, we recommend keeping the number of sterile tools as low as possible to avoid accidental contamination. Tools are supplies commonly used in our laboratories are listed in the Supplementary Material.

### Intraoperative Measures

#### Induction of Anesthesia

After the mouse has rested for at least 15 min following the transport, it is weighed and the tail is marked with a marker pen for easier identification, when multiple mice are housed in the same cage. Having a calm subject during the induction of anesthesia increases the chances of stable anesthesia. Mouse is lifted from the cage by the proximal tail. The body is immediately rested on a flat surface or the palm or the back of the opposite hand of the researcher since tail suspension is stressful for the mice (Yapıcı-Eser et al., 2018) If the mouse is going to receive inhalation anesthesia, it is slowly placed in an induction chamber that is not pre-filled with anesthetic. Anesthesia is induced with isoflurane at 3% followed by 1–1.5% maintenance. Alternatively, ketamine-xylazine (K/X) injection (i.p.) can be used for induction and can be supplemented with extra doses of ketamine throughout surgery, or isoflurane. K/X has been used as a standard anesthetic for surgical procedures, but the short half life makes it less practical for longer procedures (Jaber et al., 2014). On the other hand, isoflurane anesthesia is reported to increase brain edema (Thal et al., 2012), but in our protocols with the use of preoperative dexamethasone, we have not experienced significant brain edema.

#### Surgical Procedure

The mouse is placed on the heating blanket and secured in the stereotaxic frame (please see the Supplementary Material). A sterile ointment is applied to the eyes. The application of this ointment can be repeated as often as necessary. Not only does this ointment protects the eyes from keratitis, it also protects against accidental exposure to povidone-iodine solution (P-I) and alcohol. For many behavioral experiments, facial whiskers play an important role for sensing and task performance. The whiskers can be weighed down with some lubricant so that they are not accidentally cut during surgery.

Cefazolin and buprenorphine are injected. Hair is removed with depilatory cream, and the skin is wiped with wet surgical sponges to remove remaining hair. The bare skin is cleaned with a 5% P-I solution (e.g., Betadine) followed by alcohol swabs repeatedly for three times with alternating wipes. P-I solution should be completely rinsed off since it may cause the mouse to itch when dried up. The skin is marked with a semi-permanent marker (e.g., fine tip Sharpie) to define the surgical borders slightly smaller than the intended size, because the skin will stretch after the cut, which makes the incision larger. The skin is cut with a #11 blade and scissors following the markings. Sub-cutaneous tissue is dissected with scissors. The remaining subcutaneous tissue is pushed aside with sterile cotton tip applicators starting from the middle and moving toward the edges until the bone is dry. The periosteum is removed by scratching with a #15 blade. This procedure is repeated until no loose connective tissue is left. A crosshatch pattern is carved on the skull with a blade sparing the exposure and surroundings to improve adherence. The skull is dried completely with pressurized air and covered with cyanoacrylate glue (Loctite 4014). For better control, a drop of glue is placed in the middle of the skull and dragged to the edge of the skin with a sterile wooden applicator (the handle of sterile cotton tipped applicator could be used after sharpening the tip with a sterile blade). It is best to position the skin in place before the application of the glue and drag the glue just until the skin edge to attach it in place.

The exposure is marked using a semi-permanent marker (e.g., fine tip Sharpie). Marking the exposure on dried glue has the advantage as the marking can be cleaned with a sterile cotton tip applicator and alcohol before drawn again for adjustments to the drawing. Head bar is attached with glue (Loctite 401) and dental acrylic. Applying the acrylic before the glue completely dries promotes a chemical reaction between the two yielding a stronger bond (Winkler et al., 2006). The details for the adjustments and attachment of different head bars are described in the Supplementary Material. When the head bar is secured in its place, the bone on the exposure is thinned along the marked craniotomy perimeter using a surgical drill and tungsten carbide burrs with 0.3-0.5 mm diameter. It is important to drill slowly (up to 15,000 rpm) and softly to prevent heating of the bone which would exaggerate inflammatory processes. A standard craniotomy takes about 15–30 min. The drill bit should be regularly dipped in chilled saline to avoid excessive heating. The bone should regularly be chilled with chilled saline prior to removal. The bone dust should be removed by washing the area with sterile saline. The excess liquid could be removed from the area using sterile wipes. Alternatively, a vacuum aspiration system equipped with a sterile blunt tip could be used. In case of bleeding from the bone, sterile sponges in saline and bone wax are utilized. It is recommended to stop drilling until the bleeding is completely under control. The thickness of the bone along the drilled perimeter can be checked by pressing on the bone gently. When the bone is thin enough along the drilled perimeter, the central piece is easily depressed when gently pushed. Prior to removal of the central piece, a drop of saline at 37° C is placed on the exposure. Using a craniotomy forceps, gently check for an edge which can be pierced. Slowly pry the edge and follow around. It is important to proceed slow to avoid bleeding from the dura. When the bone is loose enough, tear the remaining edge and remove the bone. At this point a piece of saline-soaked gel foam may be used to keep the dura moist, promote coagulation, and remove small residues of bone dust, and blood clots.

When all the bleeding is under control, the borosilicate glass or polymer is placed on the exposure replacing the bone. The window is pushed down and inside the craniotomy such that the bottom surface of the window comes in touch with the brain surface. A stereotaxic frame manipulator equipped with a sterile plastic pipette tip or a sterile wooden stick can be used to hold the glass in place. For soft polymer windows, manual handling may be needed. The details for the attachment of specific glass or polymer windows is described in the Supplementary Material. The cover is then sealed with glue, dental acrylic, and a second layer of glue. The whole skull surface and the part of the head bar that is attached to the skull are covered with dental acrylic and a thin layer of glue. 0.1 ml of 5% dextrose is injected subcutaneously for surgeries up to 2 h. If the surgery takes longer than that, this injection is repeated every 2 h.

After the glue and acrylic are completely dry and the animal is marked (e.g., by ear notch), the exposure is covered by a protective cap (please see the following section). The animal is then placed in a clean cage that is positioned on top of a heating blanket set to 37°C and is monitored until it regains motor control before being returned to the vivarium. In case of isoflurane, the mouse should regain some motor control within 10 min and eating/drinking within 1 h after a successful surgery. In case of K/X, the mouse is expected to recover some motor functions after ~30 min following the last supplemental K injection, but the time course of recovery can vary between animals.

#### Protective Caps

For chronically prepared animals we suggest using a protective cap on top of the exposure. This prevents the possible break in the glass and protects soft exposure covers like polymers.

In the case of glass windows, regardless of the head bar design, casting silicone can be used right before the end of surgery. Casting silicone components are mixed 1:1, and a large drop is applied to cover the glass windows and surrounding acrylic. This polymer helps keep the glass safe and clean and acts as a thermal insulation barrier. The anesthesia is discontinued when the silicone is set (~5 min following application). This silicone can be gently peeled for imaging sessions leaving no residue behind and can be reapplied when the session is over and before the animal is released to the home cage.

When using the polymer covers, we suggest using harder materials to cover the surface. While 3-D printed caps are the most convenient ones to use, custom machined caps can also be used. Cap can be mounted on the head bar using interlocking designs or magnets (not MRI safe). For an exemplary design please see the Supplementary Material.

### Post-operative Measures (~ <10days)

#### Monitoring

Monitor the mouse daily for 5 days following surgery assessing general appearance, weight, and pain scale (Langford et al., 2010). TMP-SMX/Ibuprofen is supplied in drinking water during this period. If there are signs of pain, additional buprenorphine injections should be given in 12-h periods for up to 3 days. It is also helpful to give some softened pellets of food immersed in medicated drinking solution or gel food or hydrogel to feed and hydrate the mouse.

#### Training for Head Fixation

Training sessions can start when the initial monitoring of the animal is completed (post-operative ~7 days). The mouse is handled until calm and trained to sit still in the cradle for up to 1 h with increasing duration (e.g., 15, 30, 45 min, 1 h). On imaging days, once the animal sits in the cradle, it is quickly fixed and left to rest for 5 min before moving the cradle under the microscope. It is rewarded with a treat (e.g., sweetened condensed milk). The cradle is positioned in the imaging setup and reward is offered about every 15 min. If the mouse shows signs of discomfort or anxiety, the session is aborted, and the mouse is released. Forceful movement and struggling can lead to the mouse detaching its head bar.

#### Imaging Under Light Anesthesia

The mouse can be imaged under light anesthesia (K/X or isoflurane) when no functional data is needed. Eyes should be protected with ointment. The glass window can be wiped with alcohol and, if necessary, it can be gently scraped with a blade. Soft polymer windows should not be treated with alcohol. Instead, sterile saline can be used. This imaging protocol is suitable for morphological imaging where small movements may cause imaging artifacts the researcher wants to avoid.

Over the course of days 1–10 after surgery, exposure may become inflamed and some dural vessels could develop from the sides, but this should resolve, and the exposure should be ready to image around day 10–14 after surgery. High-quality exposures allow imaging of intravascular fluorescent tracer fluorescein isothiocyanate (FITC)-dextran (2 MDa) down to at least 500 μm with 900 nm excitation.

### Imaging (~Day 10+)

#### Delivery of Contrast Agents

For imaging protocols that involve i.v. contrast agents, a brief anesthesia is induced with 3% isoflurane in O_2_ or air. Retro-orbital injection (Yardeni et al., 2011) of contrast agents (e.g., 0.05 ml FITC at 5% in PBS) is performed using a tuberculin syringe 31 G, 0.5″ needle. Care is taken not to scratch the orbital fossa (bone), because this may cause irritation and pain when anesthesia is discontinued. Alternatively, intravenous injections can be given through the tail vein, however, this may result in local inflammation with imperfect application and is not recommended for novice researchers.

#### Awake Imaging

In cases where an i.v. injection is performed, the mouse is allowed to wake up and recover from anesthesia for 15–60 min. The mouse is handled until it becomes calm. After that, the head bar is fixed in the mouse holder and brought in the imaging setup. Reward is offered in line with the experimental protocol. The imaging session should not last longer than 60–90 min. The mouse should be released and returned to its home cage when the frequency of its movement starts increasing. Movement can be detected via cameras and/or accelerometer (Bergel et al., 2018). The mouse is returned to its cage and the cage to vivarium after the imaging session is over.

#### Euthanasia

Whenever, the experimental study is completed or if the exposure is no longer imageable, euthanasia is performed in accordance with Institutional Animal Care and Use Committee guidelines.

## Results

In this section, we will present example imaging data that were acquired from chronically prepared animals. Details of specific surgical procedures and imaging protocols are described in the Supplementary Material.

### Glass Windows Used in 2-Photon Microscopy, IOSI, LSCI, and MRI-Compatible Optical Imaging in Conjunction With OG Stimulation

**Figure 1:**
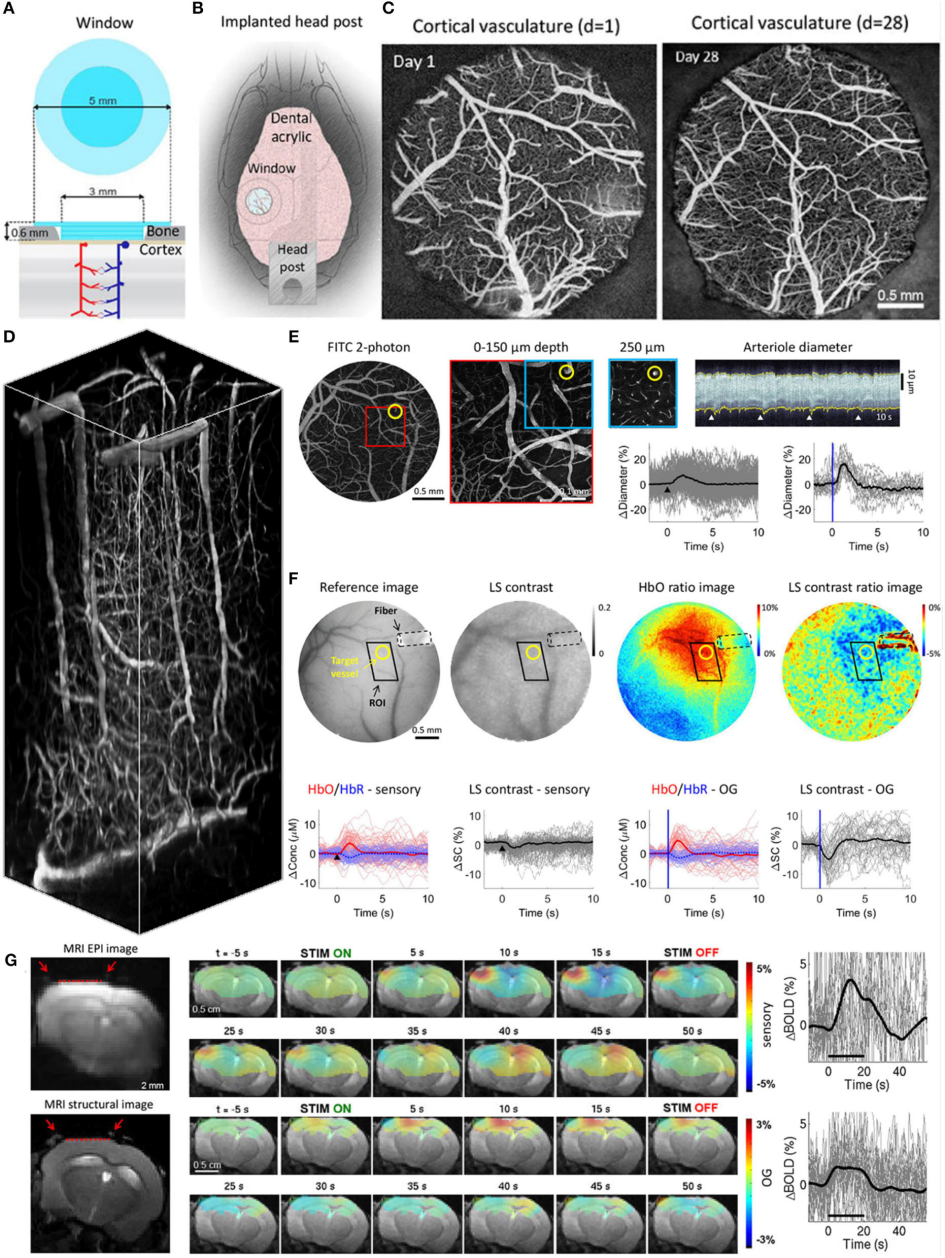
**(A)** Schematics of the borosilicate glass window implant. **(B)** Schematic illustration of the window implant over the whisker representation within the primary somatosensory cortex (SI) and the headpost fixed to the skull overlaying the other (contralateral) hemisphere. **(C)** Images of the brain vasculature through the glass window implant obtained by 2-photon imaging of fluorescein isothiocyanate (FITC)-labeled dextran injected intravenously. The images illustrate preserved integrity of the vasculature between days 1 (left) and 28 (right) following surgical implantation. **(D)** Two-photon image stack obtained with Alexa 680 labeled dextran injected intravenously illustrating the capability of deep imaging. The resolution for presented images was ~1 μm/pixel. **(E)** Top: Image of the surface vasculature calculated as a maximum intensity projection (MIP) of an image stack 0–300 μm in depth using a 4 × objective. Individual images were acquired every 10 μm (top, left). A zoomed-in view of the region within the red square acquired with a 20× objective (top, middle, left). A plane 250 μm below the surface corresponding to the region outlined in blue (top, middle, right). The yellow circle indicates a small diving arteriole. An example temporal diameter change profile acquired from the arteriole outlined by the yellow circle imaged 250 μm below the surface (top, right). The vessel diameter was captured by repeated line-scans across the vessel. These line-scans form a space-time image when stacked sequentially, from left to right. White arrowheads indicate the onset of stimulus trials (air puffs to the whisker pad); four trials are shown. Graphs: Single-vessel dilation time-courses extracted from data. Time-courses for individual trials are overlaid for sensory stimuli (left, *n* = 160 trials) and OG stimuli (right, *n* = 19 trials); the thick lines show the average. The stimulus onset is indicated by the black arrowhead and the blue vertical line for the sensory and OG panels, respectively. **(F)** Concurrent IOSI and LSCI in the same subject. A CCD reflectance image of the surface vasculature (left). The corresponding LS contrast image (middle, left). Ratio images of HbO (extracted from the OIS data, see Methods) and LS contrast showing the region of activation following OG stimulation (middle, right, and right). The location of optical fiber is indicated on all images (black dotted line). The same arteriole is outlined by yellow circles. The black parallelogram indicates the region of interest (ROI) used for extraction of time-courses. The resolution for presented images was 5.5 μm/pixel. Graphs: Time-courses of HbO and HbR (shown in red and blue, respectively), and LS contrast (shown in black) in response to sensory and OG stimulation. These time-courses were extracted from the polygonal ROI shown in top images. **(G)** Corrected GE EPI image (left, top) and a corresponding structural image (TurboRARE, left, bottom). The resolution for EPI and TurboRARE is 200 μm/pixel 100 × 50 and ~75 μm/pixel, slice thickness = 1 mm. Red arrows point to the peripheral edges of the implant, i.e., the glass/bone boundary. The red line indicates the bottom of the glass implant, i.e., the glass/brain boundary. The BOLD signal in response to sensory stimuli in a fully awake mouse (top, middle). Spatiotemporal evolution of the BOLD signal change from a single slice cutting through the center of the evoked response, presented as trial-averaged ratio maps, in response to a 20-s train of 100-ms light pulses delivered at 1 Hz (“blocked” OG stimulus) in a single Emx1-Cre/Ai32 subject. EPI images were thresholded to reflect the sensitivity of the surface RF coil (for display purposes only). The ratio images are overlaid on the structural (TurboRARE) image of the same slice. BOLD response time-courses extracted from the active ROI (top, right). Fifty seven stimulus trials are superimposed. The average is overlaid in thick black. The BOLD signal in response to OG stimuli in a fully awake mouse (bottom, middle). Spatiotemporal evolution of the BOLD signal change from a single slice cutting through the center of the evoked response, presented as trial-averaged ratio maps, in response to a 20-s train of 100-ms light pulses delivered at 1 Hz (“blocked” OG stimulus) in a single Emx1-Cre/Ai32 subject. EPI images were thresholded to reflect the sensitivity of the surface RF coil (for display purposes only). The ratio images are overlaid on the structural (TurboRARE) image of the same slice. BOLD response time-courses extracted from the active ROI (bottom, right). Twenty eight stimulus trials are superimposed. The average is overlaid in thick black. Modified from Desjardins et al. (2019).

This preparation is a modification of the design described by Chen et al. (2015). It has been used by our group in several studies including Desjardins et al. (2019). A glass plug constructed from one 5-mm round coverslip and two or three 3-mm round coverslip is used in this surgery (Figures 1A,B). Structural imaging demonstrating the state of the surface vessels 1 and 28 days after the surgery is obtained after i.v. (retroorbital) injection of FITC-dextran (Figure 1C). The quality of the window can be further appreciated in a 3D vascular stack that was obtained after injecting Alexa 680-dextran (Figure 1D). Functional data were obtained with 2-photon imaging of vasodilation in response to sensory stimuli applied to the whiskers as well as OG stimuli stimulation in animals expressing VGAT-ChR2 (Figure 1E). The same animals can be used for measurements of oxygenated hemoglobin (HbO), reduced hemoglobin (HbR), and total hemoglobin (HbT) concentrations using IOSI and cortical blood flow using LSCI in response to sensory and OG stimuli (Figure 1F). Finally, the same animals can be used for optical imaging and OG stimulation performed simultaneously with fMRI when headposts are made from polyether ether ketone (PEEK) plastic (Figure 1G). For details, please see the Supplementary Material and Desjardins et al. (2019).

We have also modified these glass windows with polymer sealed ports for injection of drugs or various fluorophores (Roome and Kuhn, 2014). These ports also allowed insertion of thin electrodes or optical fibers. Please see the Supplementary Material for details.

### Half Crystal Skull Covered Craniotomy Used in 2-Photon Microscopy, IOSI, LSCI, and OCT Imaging to Evaluate the Effects of Stroke Caused by Photothrombosis

**Figure 2:**
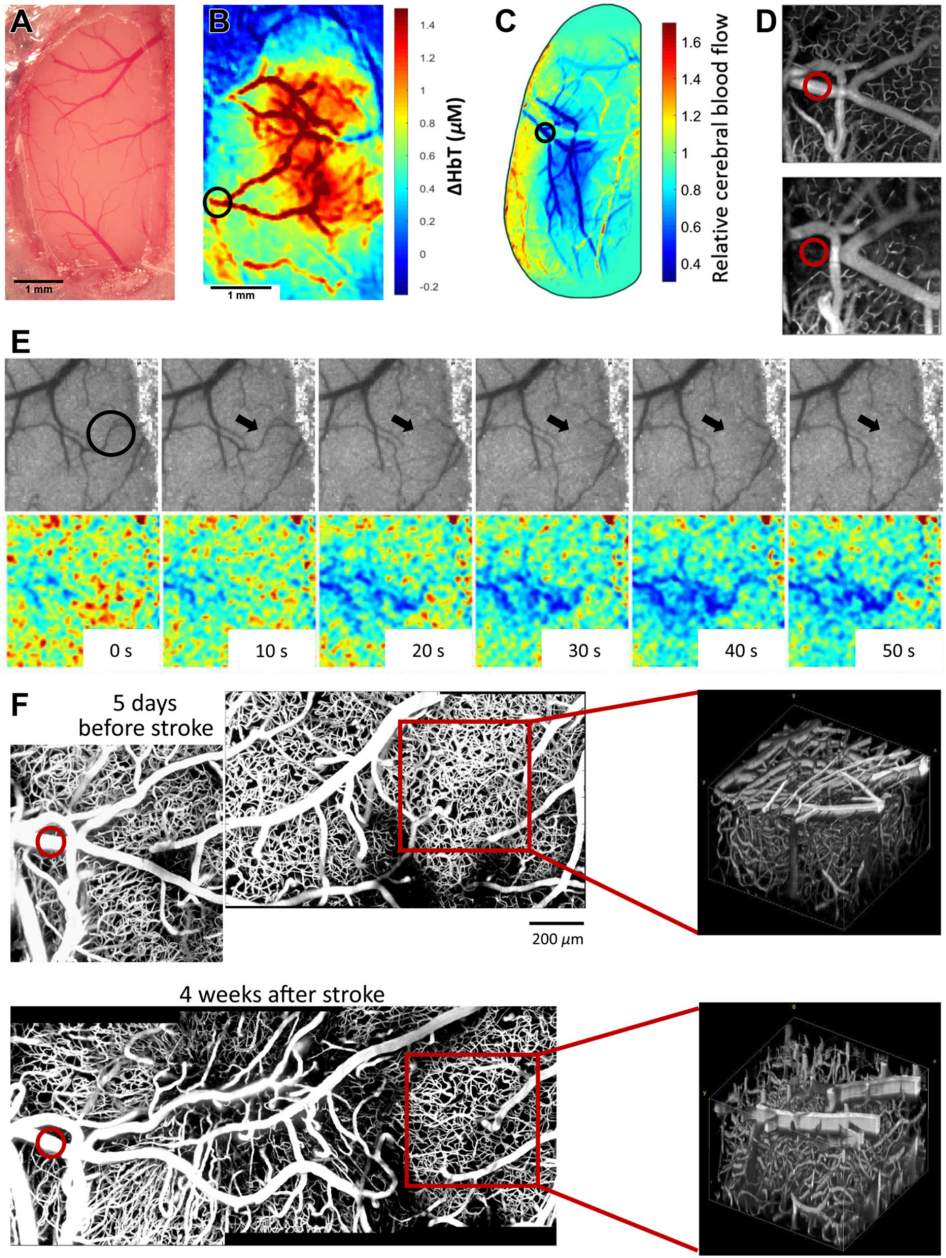
**(A)** Representative image of cranial window immediately after surgery. In this preparation, animal was implanted with a one-point secured flat head bar and half Crystal Skull glass (please see Supplementary Material for details). **(B)** Intrinsic optical signal imaging of change in total hemoglobin concentration during air puff stimulation of the contralateral forelimb. Black circle indicates the vessel targeted for photothrombotic occlusion. The resolution for presented images was 5.5 μm/pixel. **(C)** Relative CBF detected by LSCI at 1 h after photothrombosis. The resolution for presented images was 5.5 μm/pixel. **(D)** OCT angiograms of flowing vessel before stroke (top) and 1-h after photothrombotic stroke (bottom). Transverse and axial resolutions of the OCT system using a 10× objective (Mitutoyo) were 3.5 and 3.5 μm. **(E)** Representative images showing collateral occlusion (circled in black). Top panel shows laser speckle contrast images as visualized in real time. Bottom panel shows relative blood flow changes associated with the occlusion. **(F)** Two-photon maximum intensity projections (left) and volumes (right) of 400 μm stack 5 days before photothrombosis (top) and 4 weeks after photothrombosis. Red circle indicates vessel targeted for photothrombosis. Red square indicates regions chosen for volume projections. The resolution for presented images was ~1.5 μm/pixel. Modified from Sunil et al. (2020).

This preparation is a modification of the design described in Kim et al. (2016) where they have created a curved glass replacement to dorsal cranium and termed it the “Crystal Skull.” We have modified this protocol using half of this commercially available curved glass (labmaker.org) to prevent the disruption of blood flow in sagittal sinus while extending the coverage to visualize the distal branching points of MCA and the entirety of barrel cortex which are more laterally positioned than the original design of Crystal Skull placement. We have used our version of preparation in several studies including Sunil et al. (2020). In cases where a large cortical area is desired to be imaged, surgery using crystal skull glass can be performed (Kim et al., 2016). However, due to high risk of bleeding or thrombosis, researchers may wish to avoid preparations involving the sagittal sinus. We have successfully implemented a modified procedure that allows us to image one hemisphere, which is sufficient for our application (Figure 2A). This preparation prevents drilling over the sagittal sinus, and small displacement of the glass in the lateral direction allows covering the hemisphere up to the lateral ridge, which cannot be achieved with the original method. Here we present results from experiments involving IOSI (Figure 2B), LSCI (Figures 2C,E), OCT (Figure 2D), and 2-photon microscopy (Figure 2F). We leveraged these large windows in studies of recovery from experimentally induced photothrombotic stroke in chronic, awake animals. The half crystal skull window allowed us to longitudinally image the stroke area after distal middle cerebral artery (Figures 2B–D,F, circles) and collateral occlusion (Figure 2E, arrow). Both the stroked and surrounding healthy tissue (Figure 2F) was accessible in the same animal for a month after the stroke was induced, allowing for long-term assessment of the pathology. For details, please see the Supplementary Material and Sunil et al. (2020).

### Soft Polymer Window Used in 2-Photon Microscopy, IOSI, LSCI, OCT, and fUS Imaging

**Figure 3:**
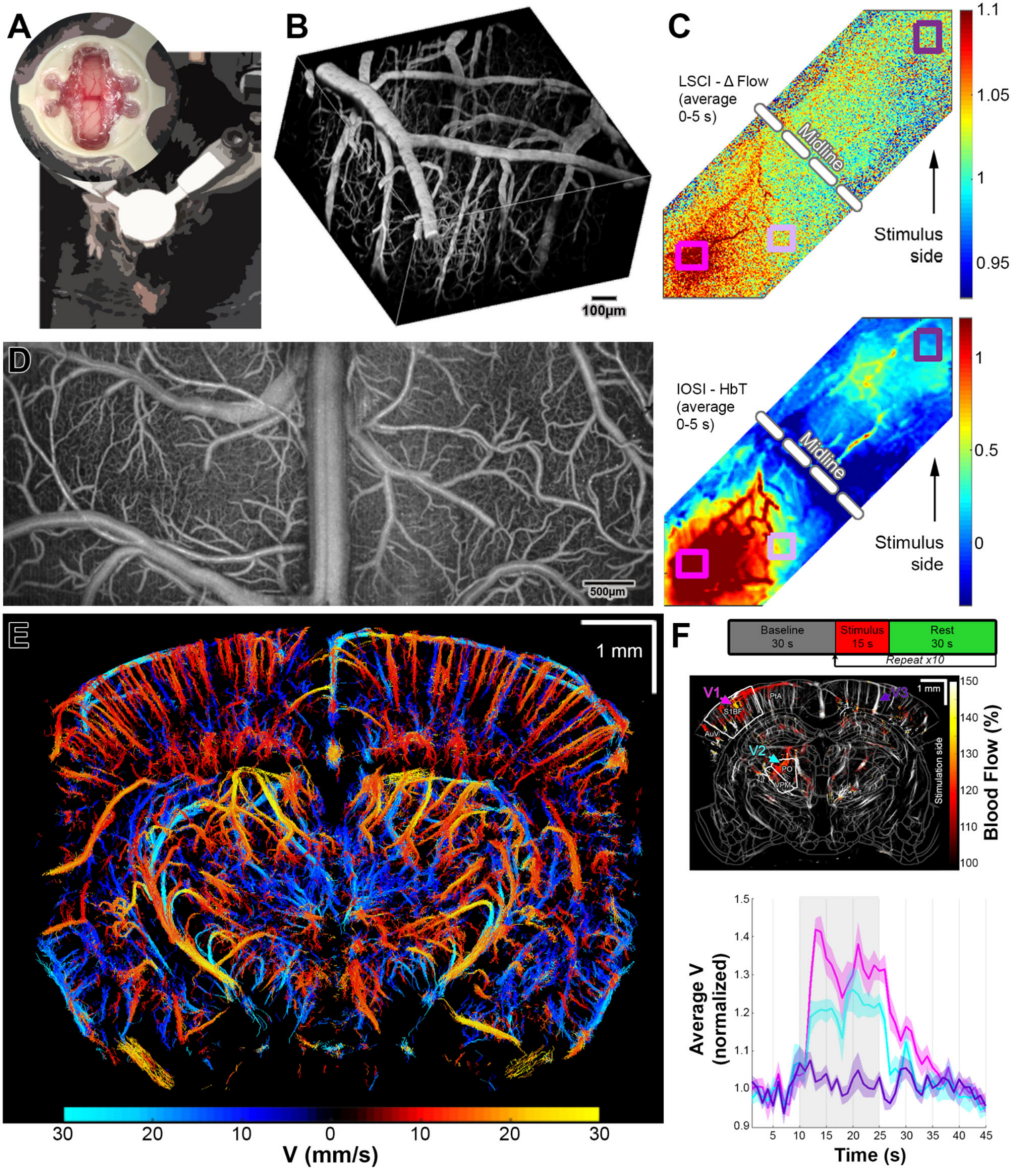
**(A)** Representative images of US compatible craniotomy (top) and the position of an awake mouse for training and imaging. In this preparation, animal was implanted with a two-point secured machined head bar and a PMP strip (please see Supplementary Material for details). **(B)** 2-P vascular stack taken with i.v. FITC injection. The resolution for presented images was ~1.5 μm/pixel. **(C)** LSCI (top) and IOSI (bottom) of both hemispheres during whisker stimulation. The resolution for presented images was 5.5 μm/pixel. **(D)** Full field OCT angiography. Transverse and axial resolutions of the OCT system using a 5× objective (Mitutoyo) were 7 and 3.5 μm. **(E)** ULM images acquired by US. The resolution for presented images was ~10 μm/pixel. **(F)** Experimental paradigm for fUS imaging (top). Trial average of fUS images acquired during whisker stimuli (middle). The resolution for presented images was ~100 μm/pixel. Time series of average velocity values calculated for marked vessels (middle) during whisker stimuli. Note that contralateral cortical and subcortical activation is visible while ipsilateral cortex is not activated. Modified from Tang et al. (2020).

This preparation is a modification of the “soft window” design described in Boido et al. (2019). We used this preparation in several studies including Kılıç et al. (2020) and Tang et al. (2020). Although glass craniotomies are preferred because of the durability, glass has properties incompatible with US imaging. Here, we show typical results from animals prepared with a polymethypentene (PMP) polymer soft window (Figure 3A). This type of window is compatible with 2-P microscopy (Figure 3B), LSCI and IOSI (Figure 3C), OCT (Figure 3D), ultrasound localization microscopy (ULM, Figure 3E), and fUSG (Figure 3F). In contrast with the soft windows made from PDMS (Heo et al., 2016), PMP windows are less permeable to air and have no air bubbles formed on the cortical surface which makes them more suitable for US imaging since the air bubbles will lead to loss of signal. Although these windows are very stable, compared to glass counterparts, the optical imaging quality may degrade faster over time. For example, 2-P microscopy imaging at 800 nm with FITC-dextran in a glass preparation, can acquire good images down to 500–600 μm in cortex with quality comparable in first and the sixth month. However, with PMP windows, while penetration in the first month with the same imaging modality is comparable, we have experienced that we could only image 300–400 μm deep after 6 months and 150–200 μm after 1 year (data not shown). For details, please see the Supplementary Material and Kılıç et al. (2020) and Tang et al. (2020).

## Discussion

The surgical procedures described in this manuscript and the Supplementary Material are easy to follow for an experienced surgeon. For novice surgeons, we recommend starting surgical training using healthy, wild type, young adult mice (8–12 weeks). For the first training sessions, avoiding the sutures between skull bones will increase the success rate. During training, a surgeon also gets accustomed to a varying bone thickness across the dorsal surface of the mouse skull. After the goals of this training are achieved, craniotomies crossing the midline may be attempted. Here, the main challenge is to meticulously remove the bone without damaging the underlying sagittal sinus. Even for experienced surgeons, using two half crystal skull windows instead of making a single bilateral exposure may be preferable. This is because exposing the midline entails high risk of bleeding and thrombosis of the sagittal sinus.

The window quality depends not only on the optimization of the craniotomy procedure but also on the proficiency in sealing the window. This step may take some time to master. Once all the procedures are mastered, a mouse with a chronic window implant can be imaged routinely up to 6 months with good outcome, although instances where imaging was performed for up-to 1.5 years after surgery have been reported (Füger et al., 2017).

Researchers should keep in mind that inflammation peaks around the third day post-operatively and usually takes a few weeks to resolve. While the inflammatory processes are ongoing, the tissue will remain opaque and not suitable for optical imaging. Therefore, imaging should start around 3 weeks after the surgery, although training sessions can start earlier but not within the first week after the surgery. In our experience, the best efficiency is achieved when the mouse is given 1 week to recover before starting the training and at least 4 training sessions in the upcoming 10–14 days before starting the imaging. The exact schedule may vary slightly depending on the subject.

Lastly, performing a craniotomy can be more invasive compared to the thinned skull preparations (Shih et al., 2012); but may give more flexibility with different imaging modalities. Removing the skull completely eliminates a problem of inconsistent thickness of the thinned skull preparations, provides less scattering and therefore deeper optical penetration. Also, most thinned skull preparations (Drew et al., 2010; Shih et al., 2012) require the used of reinforcement which makes them not suitable or less desirable for some imaging methods like fUS or for optical imaging performed simultaneously with MRI.

Although truly simultaneous application with all of the imaging/stimulation techniques mentioned here is not feasible, one mouse can rotate between imaging instruments, sometimes on the same day, provided that it is given enough time to rest in between imaging sessions in the home cage. Some of the abovementioned imaging modalities can be readily combined in a multimodal setup that allow simultaneous use of the systems. Examples include (1) LSCI, IOSI and photothrombosis (Kazmi et al., 2013; Sunil et al., 2020) (2) 2-P microscopy and optogenetic stimulation (Bovetti et al., 2017; Yang et al., 2018), and (3) MRI and optical imaging and/or optogenetic stimulation (Lin et al., 2016; Schlegel et al., 2018; Chen et al., 2019).

## Conclusion

Even though it was introduced around 60 years ago, the 3R (Replacement, Reduction, Refinement) principle is exceedingly relevant and important to the current *in vivo* data acquisition protocols (De Angelis et al., 2019). These principles serve as gatekeepers on the way of improving both the quality of life of the animals used, as well as the quality of the data collected. The surgical procedures described here are designed to maximize the spectrum of imaging techniques that can be applied on the same subject. They are also modified to decrease the complications after surgery, morbidity, and mortality therefore decreasing the number of the experimental subjects.

We hope these detailed protocols will be helpful for increasing the rigor and repeatability in imaging studies and decreasing the number of animals utilized by these studies.

## Data Availability Statement

The raw data supporting the conclusions of this article will be made available by the authors, without undue reservation.

## Ethics Statement

The animal study was reviewed and approved by UCSD and Boston University. Some parts of this study are conducted at University of California, San Diego (UCSD).

## Author Contributions

KK: development of surgical methods, acquisition of data, analysis of data, and writing the manuscript. MD, MT, SS, and ŞE: acquisition of data, analysis of data, and writing the manuscript. JT and DP: development of imaging algorithms, acquisition of data, analysis of data, and writing the manuscript. DB and AD: supervising the experiments and writing the manuscript. All authors contributed to the article and approved the submitted version.

## Conflict of Interest

The authors declare that the research was conducted in the absence of any commercial or financial relationships that could be construed as a potential conflict of interest.
