# Supplementary material for: Chronic Cranial Windows for Long Term Multimodal Neurovascular Imaging in Mice
Source: Front Physiol. 2021 Jan 22;11:612678. doi: 10.3389/fphys.2020.612678 (PMC7862556; doi:10.3389/fphys.2020.612678)
Supplement: Supplementary file 1 [file Data_Sheet_1.pdf]

Supplementary Material

Chronic Cranial Windows for Long Term Multimodal Brain Imaging in Mice

Kıvılcım Kılıç[1,2,*], Michèle Desjardins[1,3], Jianbo Tang[2], Martin Thunemann[1,2], Smrithi Sunil[2], Şefik Evren Erdener[2,4], Dmitry D. Postnov[2], David A. Boas[2,5] and Anna Devor[1,2,5]

1 University of California, San Diego, La Jolla, CA, United States of America; 2 Boston University, Boston, MA, United States of America; 3 Université Laval, QC, Quebec, Canada; 4 Hacettepe Üniversitesi, Ankara, Turkey; 5 Martinos Center for Biomedical Imaging, Massachusetts General Hospital, Harvard Medical School, Charlestown.

* Corresponding author

In this supplementary material we detail the protocols and techniques used by our group that are presented in the manuscript. Any experiments using animals must be conducted in accordance with the institutional and national guidelines and regulations.

TABLE OF CONTENTS

1. Materials
   a) Tools
   b) Supplies
   c) Surgical Equipment
   d) Optional
2. Perioperative Measures
3. Techniques
   a) Stereotaxic Frame Placement:
   b) Attachment of Head Bars:
      i- One point secured flat head bars
      ii- Two point secured machined head bars
      iii- Three point secured magnetic head bars
   c) Craniotomy Procedures
      i- Small Round Craniotomies (3-5 mm)
         ♦ 3 mm Round Craniotomy for Barrel Cortex
         ♦ 4-5 mm Round Craniotomies
         ♦ 3mm Glass Plug with Port
      ii- Half Crystal Skull (CS) Craniotomies
         ♦ Cutting and Shaping the CS glass
         ♦ Single Sided (unilateral)
         ♦ Double Sided (bilateral)
      iii- Bilateral Strip Craniotomies
      iv- Reflective Prism Insertion
      v- Intracerebroventricular (icv) Cannula Insertion
      vi- Making the Imaging "Well"
      vii- When Things Go Wrong
4. Supplementary References

1. MATERIALS

a) Tools:

In this section, we describe the use of some basic tools for common surgeries. Specific tools are listed under specific procedures. We recommend keeping the number of tools low to avoid clutter. Non-sterile tools should be placed on the table, not on the sterile tray. Tools and their uses are summarized in Table 1.

Tool	Sterile	Uses
One pair of soft tissue forceps (e.g. Adson)	Yes	To hold skin and subcutaneous tissue
One pair of extra fine Bonn scissors	Yes	To dissect the skin and subcutaneous tissue
Two blade handles (with #11 and #15 blades)	Yes	#11 to cut the skin and 15 to scratch the periosteum
One pair of laminectomy forceps	Yes	To lift the bone flap
One pair of Mayo scissors	No	To cut non-sterile sponges, tape, etc.
One pair of soft tissue forceps	No	To position the tongue, paws, etc. of the animal
Pliers (optional)	No	To remove the metal ring from the sterile saline bottles, to bend the metal head bars

Table 1. Common tools for surgeries

b) Supplies:

- Eye ointment
- Lubricant (for placement of the rectal temperature probe)
- Buprenorphine filled syringe
- Cefazolin filled syringe
- Dextrose (5%) filled syringe
- Water (to facilitate hair removal)
- Hair removal cream
- Non-sterile gauze (for hair clean up)
- Non-sterile cotton tipped applicators
- Betadine wipes
- Alcohol wipes
- Marker (Fine tip Sharpie)
- Sterile cotton tipped applicators
- Glue (Loctite 401 and 4014)
- Dental acrylic
- Surgifoam (sterile) in saline
- Kim wipes
- Sterile saline (room temp and chilled)
- Sterile glass pipette
- Bone wax
- Glass or polymer for covering the craniotomy
- Casting silicone (Smooth-On Body Double Standard)

c) Surgical Equipment:

- Small animal stereotaxic frame
- Homeothermic blanket
- Bead sterilizer
- Steromicroscope
- Surgical light source
- Anesthesia delivery system equipped with scavenger

d) Optional:

- razor blades
- Ketamine- xylazine anesthesia (K/X)

2. PERIOPERATIVE MEASURES

Dexamethasone is used to prevent cerebral edema during and following the surgery (Hedley-Whyte ET, *et al*). Dexamethasone is injected intraperitoneally 4-6 hours before the craniotomy.

For short surgeries or imaging sessions (up to 30 minutes) K/X anesthesia may be preferred. If the procedure takes longer than 30 minutes, a second dose may be needed. It is recommended that xylazine should only be used for the first injection while subsequent injections should consist of ketamine alone (Green CJ, *et al*). Please see Table 1 for recommended doses. For longer surgeries, isoflurane delivered with a precision vaporizer is preferred for better control of anesthesia depth. In mice, isoflurane is usually induced by 3-4% using an air-tight induction chamber, followed by a maintenance dose of 1-1.5%.

The opioid analgesic Buprenorphine is injected right after induction of anesthesia. Buprenorphine is diluted to a final concentration of 0.03 mg/ml in saline. It is injected subcutaneously with addition of 0.1 ml (per 25 g animal) 5% dextrose solution to prevent dehydration during surgery (Schuler B *et al*). Buprenorphine is injected every 12 hours if signs of pain or discomfort are observed (Langford DJ, *et al*). Recently, a slow-release formulation of Buprenorphine became available providing analgesia over a 72-h period with a single injection (Foley PL *et al*.). Cefazolin is injected as prophylactic antibiotic (Liang P, *et al*). It is injected intraperitoneally at a final concentration of 200 mg/ml in saline. For injectable drug doses please see Table 1.

A mixture of the antibiotics trimethioprim and sulfametoxazole (TMP-SMX) and the non-steroidal anti-inflammatory drug ibuprofen (I) can be used for pre- and postoperative treatment. Therefore, a suspension of TMP (8 mg/ml), SMX (40 mg/ml), and I (20 mg/ml) is prepared in drinking water and administered orally from 24 hours before through five days after surgery. The suspension needs to be agitated daily to prevent settling. Alternatively, NSAIDs (such as meloxicam or ketoprofen) can be given by intraperitoneal injection.

	Dexamethasone (4 mg/ml, 4.8 mg/kg)		Ketamine (40 mg/ml, 100 mg/kg) / Xylazine (4 mg/ml, 10 mg/kg)		Cefazolin (200 mg/ml, 0.5 g/kg)		Buprenorphine (0.03 mg/ml, 0.05 mg/kg)	
Mouse weight (g)	Dose (mg)	Volume (ml)	Dose (mg)	Volume (ml)	Dose (mg)	Volume (ml)	Dose (mg)	Volume (ml)
20	0.096	0.03	2 / 0.2	0.05	10	0.05	0.001	0.03
25	0.120	0.03	2.5 / 0.25	0.06	12.5	0.06	0.00125	0.04
30	0.144	0.04	3 / 0.3	0.08	15	0.08	0.0015	0.05
35	0.168	0.04	3.5 / 0.35	0.09	17.5	0.09	0.00175	0.06
40	0.192	0.05	4 / 0.4	0.10	20	0.10	0.002	0.07
45	0.216	0.05	4.5 / 0.45	0.11	22.5	0.11	0.00225	0.08
50	0.240	0.06	5 / 0.5	0.13	25	0.13	0.0025	0.08

Table 2. Drug Doses

3. TECHNIQUES

a) Stereotaxic Frame Placement:

When placing the mice in the frame, make sure to position the ear bars first and then the teeth and the nose. When both ears are placed, the distances on the ear bars should read the same ensuring that the head is in the middle. Top incisors need to be placed in the front notch of the frame and the notch should be slowly positioned farther to reach the maximum distance. After this, the nasal bone should be secured with the top attachment of the frame. At this point, when you press on the head, it should be extremely stable. If you feel any movement, release the mouse and place it again since any movement during surgery will decrease your chances of a good procedure. The top of the head should be parallel to the ground. This can be later confirmed by making sure that bregma and lambda are on a parallel plane.

b) Attachment of Head Bars:

The technique to attach a head bar depends on the design of the head bar. Here some head bar examples used by our group are given and how to attach them is explained.

i- One point secured flat head bars

Design Credit: Céline Mateo
Material: Aluminum
Advantages: Easy to manufacture, inexpensive
Disadvantages: Susceptible to motion artifacts, may break if curved multiple times.

These head bars can be used with craniotomies spanning one hemisphere (Please see Supplementary Figure 1).They can be curved to fit the shape of the skull using pliers and can be autoclaved since they are made of aluminum. It is a great option for large field of view imaging experiments where the motion artifacts are not aberrant.

After the removal of the scalp and periosteum, head bar is glued to the skull and further secured with dental acrylic.

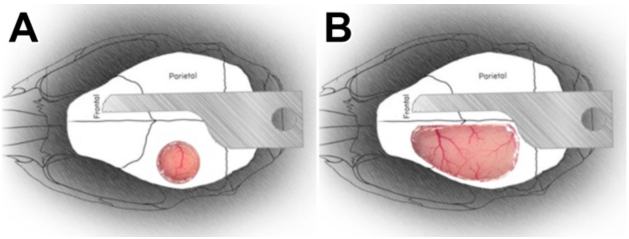

Supplementary Figure 1. One point secured flat head bars. A. with 3 mm craniotomy B. with half Crystal Skull.

*** The thin bar of the head bar should be placed parallel to the sagittal suture (the line connecting lambda and bregma) and the hole in the head bar should be in line with this suture.

ii- Two point secured machined head bars
Design Credit: Anderson Chen, John T. Giblin
Material: Stainless steel, titanium or PEEK
Advantages: Less susceptible to motion artifacts, designed to fit an adult mouse skull
Disadvantages: Extensively machined, more expensive than flat head bars

These head bars can be used with craniotomies in one or both hemispheres. (Please see Supplementary Figure 2). They are curved to fit the shape of the skull and can be autoclaved since they are mostly made of metal. It is more resistant to motion artifacts since it has two points of attachment to the cradle.

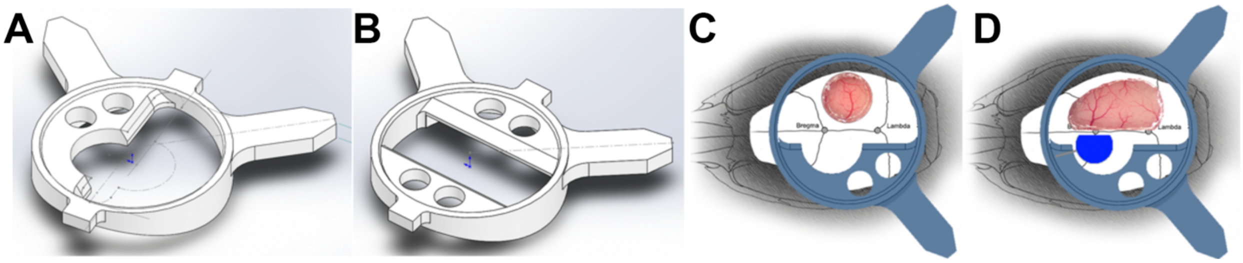

Supplementary Figure 2. Two point secured machined head bars. A. Design for left hemisphere craniotomy. B. Design for bilateral craniotomy. C. with right 4 mm craniotomy. D. with right half Crystal Skull and left icv cannula.

After the removal of the scalp and periosteum, head bar is glued to the skull and further secured with dental acrylic.

*** The attachment of these head bars requires the detachment of temporalis muscles bilaterally to increase the adherence surface. The front and the back pins should be in line with the sagittal sinus. The holes in the design are added so that the glue and acrylic under the head bar could be in contact with the glue and acrylic on the head bar to yield a higher stability.

For our design drawings, please contact the corresponding author.

iii- Three point secured magnetic head bars

Design Credit: Kıvılcım Kılıç, Blaire Lee, Su Jin Kim
Material: Stainless steel
Advantages: Less susceptible to motion artifacts, magnets make the attachment and detachment easier
Disadvantages: Slightly bigger than the other head bars

These head bars can be used with craniotomies in one or both hemispheres. (Please see Supplementary Figure 3).They can be autoclaved since they are made of metal. They are more resistant to motion artifacts since it has three points of attachment to the cradle. After the removal of the scalp and periosteum, head bar is glued to the skull and further secured with dental acrylic.

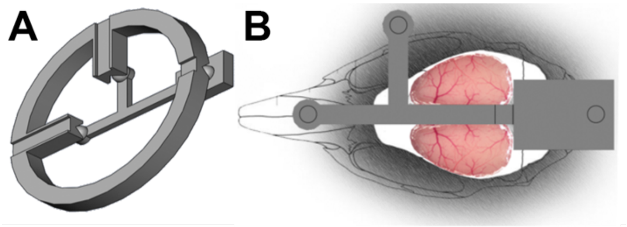

Supplementary Figure 3. Three point secured magnetic head bars. A. Example of a head bar holder. Diametrically magnetized magnets are glued into the grooves. B. with double half Crystal Skull exposures.

*** Since these head bars need to stay on the midline, if big craniotomies are planned to be performed, head bar attachment should be left to the very end of the surgery. They can also be attached during a short surgical session after the animal has recovered from craniotomy.

Magnets have been chosen as 3/16" diameter x 3/8" thick, nickel plated, diametrically magnetized cylindrical magnets (D36DIA) from https:www.kjmagnetics.com/

For our design drawings, please contact the corresponding author.

c) Craniotomy Procedures

The glass that will be used for covering the craniotomy acts like a guide. Simply position the glass on the skull (after marking the center coordinates for small craniotomies) and mark the skull with a fine tip marker. It is a good idea to measure multiple times (second measurement after the first pass of drilling) to avoid mistakes. For the craniotomies that will be covered with polymer, keep extra polymer cuts with slightly different thickness that will allow you to match the thickness to the removed bone.

i- Small Round Craniotomies (3-5 mm)

♦ 3 mm Round Craniotomy for Barrel Cortex

For C2 whisker, center coordinates are: 1.5-2.0 mm posterior, 3.0 mm lateral relative to bregma.

If you are using a glass plug made out of one 5 mm glass and concentric two-three (total thickness ~300μm, mimicking skull thickness) 3 mm glass, keep 1.5% agarose prepared in saline in hand. The glass plug is positioned so that 3 mm glass is on the bottom and 5 mm glass is on the top. Craniotomy should be made well-fitting to the 3 mm glass inside. When at least one side of the 5-mm glass is touching the bone firmly, pressure should be stopped to avoid breaking the glass. 1.5% agarose is used to fill the space between the bone and 5-mm glass so that the positioning of the glass would be secured, and the exposure is protected from the dental acrylic (Please see Supplementary Figure 4.).

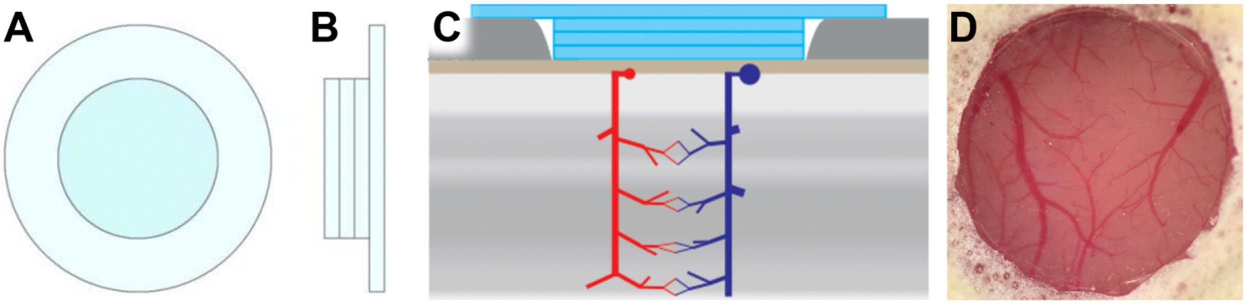

Supplementary Figure 4. 3 mm glass plug. A. Top view of plug B. Side view of plug C. Positioning of plug on brain surface and bone. D. An example craniotomy sealed with a 3 mm plug.

- **4-5 mm Round Craniotomies**

These craniotomies can be placed in the parietal or occipital cortices as a whole. It is important to make sure that the whole glass comes in contact with the brain, leaving no space between. Alternatively, the glass can be cut from one side to produce a "D" shape and be used accordingly.

- **3mm Glass Plug with Port**

These craniotomies allow parenchymal microinjections in chronic preparations. When glass plugs are prepared, the 3 mm part is slightly trimmed on the side and a hole (~300 μm in diameter) is made in the 5 mm glass. The glass is glued together using UV cured adhesive (NOA61) and the hole is sealed with a drop of clear Kwiksil. After overnight curing, the plugs are ready to use (Please see Supplementary Figure 5.).

During surgery, port is positioned to avoid big cerebral vessels. This prevents a major bleeding during injection. Injection is made using pulled Quartz pipettes with a tip of 20-30 μm. Example preparations are shown in Supplementary Figure 5 with OGB and Alexa Red injection.

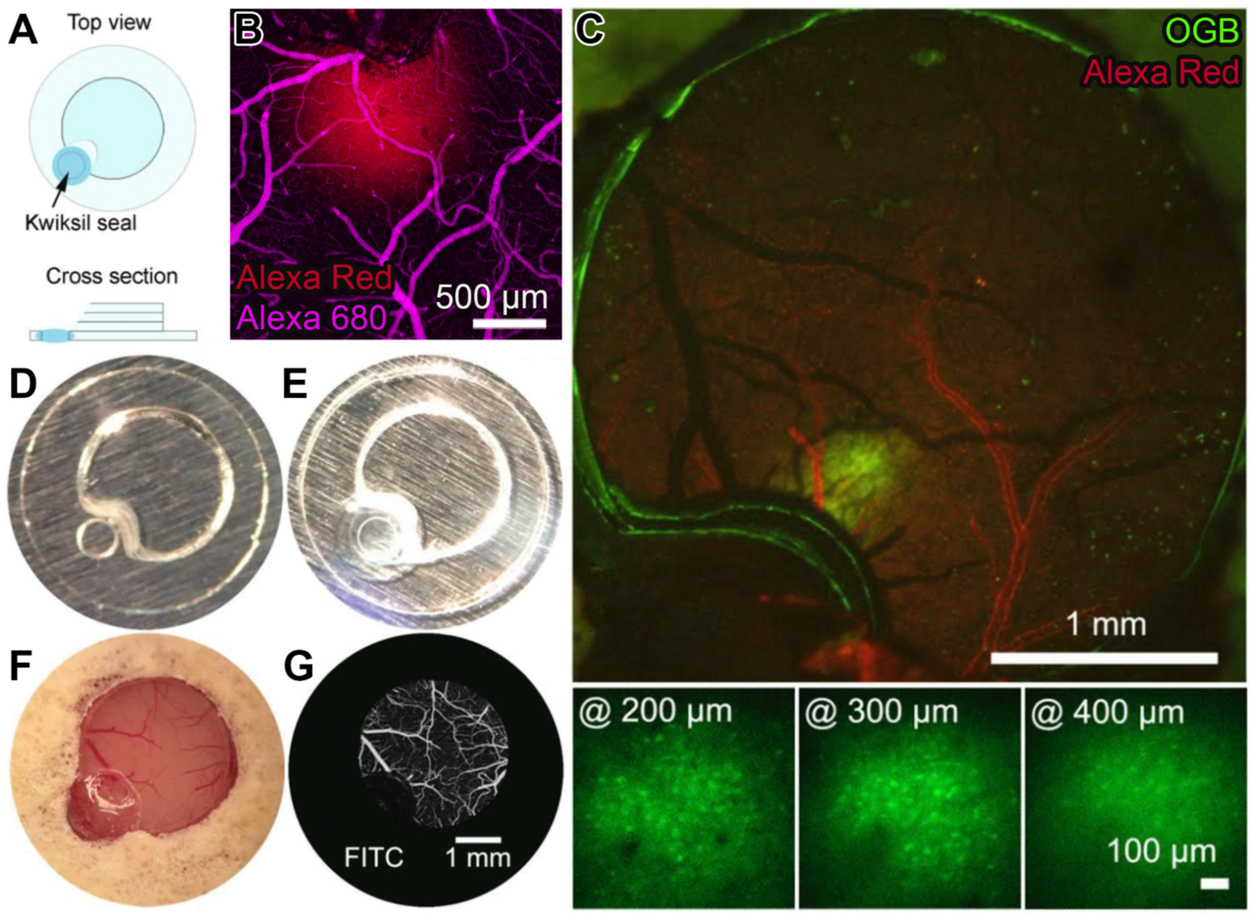

Supplementary Figure 5. 3mm Glass Plug with Port and Examples of Intracortical Injections A. Top and cross section view of the plug. B. Example of intracortical Alexa red injection with iv Alexa 680-dextran injection. C. Example of intracortical Alexa red and OGB injection and labeled neurons at different depths. D. The plug with the port open E. The plug with the port sealed F. The plug after implantation on the craniotomy. G. The MIP of vessels after iv injection of FITC-dextran.

ii- Half Crystal Skull (CS) Craniotomies

♦ Cutting and Shaping the CS glass

The CS glass can be cut in half using a razor blade and fine tip glass cutter (most common ones have tungsten carbide tips).

You need to follow the midline parallel to the axis of curvature to have a clean cut (Please see Figure 10, left panel). Without applying pressure, gently scratch the surface of the glass guided by the edge of the razor blade. You need to have at least 20 full length scratches for a clean cut. To support the glass during cutting, you can use a piece of Parafilm rolled to fit the curvature under and small pieces of tape top secure the glass. After the glass is scratched all the way, gently apply pressure to both sides of the glass (keeping the line in the middle and on the top) to crack the glass in half.

After the glass is cut in two, take one piece and mark it so that there are no sharp corners and the glass fits better to the shape of the skull (Please see Supplementary Figure 6, left panel). After the marking, place the glass between the tips of a locking forceps of those tips are covered with Parafilm, shrink tube or small tubing to avoid metal getting in direct contact with the glass. Make sure that the tips of the forceps are also placed parallel to the axis of curvature to avoid breaking the glass. Use a drill equipped with a polishing bit to shape the glass (Please see Supplementary Figure 6, right panel).

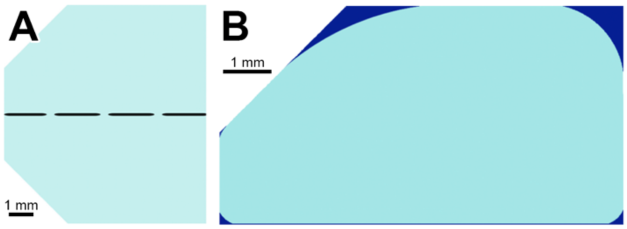

Supplementary Figure 6. Cutting Crystal Skull Glass. A. Dashed line indicates the cut. B. Blue areas indicate the trim mask.

- **Single Sided (unilateral)**

Single sided half CS craniotomies are useful when only one hemisphere need to be imaged in a large field of view (Please see Supplementary Figure 7, left panel). They also can accommodate a contralateral intracerebroventricular (icv) cannula insertion (Please see future section for icv cannula insertion and Supplementary Figure 7, right panel).

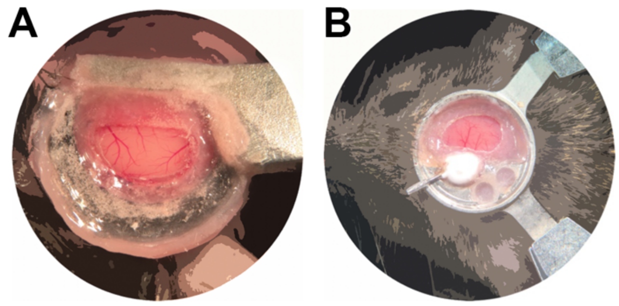

Supplementary Figure 7. Single Sided Half CS Craniotomies. A. Single sided half Crystal Skull (CS) craniotomy with one point secured flat head bar. B. Single sided half CS craniotomy with two point secured machined head bar and intracerebro-ventricular cannula.

Following general guidelines for craniotomy, after the skin incision, removing the subcutaneous tissue and periosteum, a head bar of choice is secured with glue and dental acrylic and the skull is covered with glue. If a flat head bar is used, a wide but narrow imaging well is made (please see the future section for making the imaging well). Using a fine tip marker, glass is outlined on the bone making sure that the markings do not end on the sinuses. The only suture that is safe to cross is the one between the frontal and parietal cortices. After the craniotomy is complete and bleeding control is complete (if needed) glass is placed inside the craniotomy. It is a good trick to press the glass close to the midline first and sealing only the medial edge to start with. After 60 seconds, glue will start curing and the glass can be pushed down on the lateral using the medial side as a hinge. The surroundings are then sealed with glue and acrylic.

- **Double Sided (bilateral)**

Following general guidelines for craniotomy, after the skin incision, removing the subcutaneous tissue and periosteum, a head bar of choice is secured with glue and dental acrylic and the skull is covered with glue. Using a fine tip marker, glass is outlined on the bone making sure that the markings do not end on the sinuses. The only suture that is safe to cross is the one between the frontal and parietal cortices. After one of the craniotomies and bleeding control is complete (if needed) glass is placed inside the craniotomy and sealed. The same procedure is followed for the other hemisphere. Two types of head bars can be used: midline attached, or circumference attached (Please see Supplementary Figure 8.).

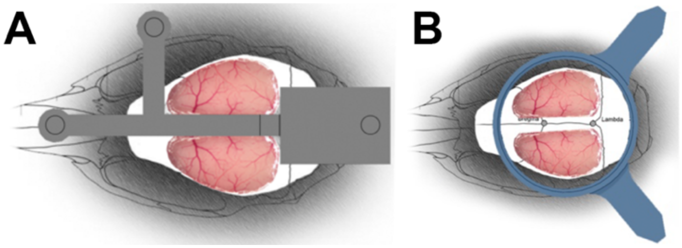

Supplementary Figure 8. Double Sided Half CS Craniotomies. A. with midline magnetic head bar B. with machined round head bar.

iii- Bilateral Strip Craniotomies

Due to the technology used by imaging equipment, craniotomy requirements may vary. For example, when imaging with ultrasonography (USG), glass cannot be used for the closure of the craniotomy. Instead, a flexible polymer needs to be used. In our USG experiments, we often use 100 μm thick polymethylpentene (PMP) which will also allow optical imaging. Since it is not possible to curve PMP in two axes, it is best to practice a strip craniotomy (Please see Supplementary Figure 9, left panel).

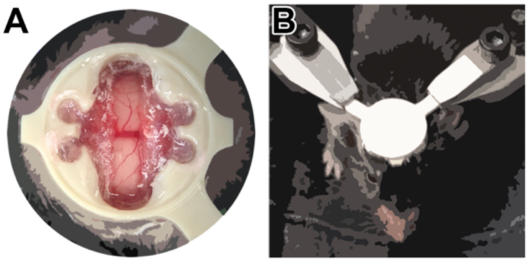

Supplementary Figure 9. Bilateral Strip Craniotomies A. Bilateral strip craniotomy with custom PEEK head bar. B. Exposure is covered when the brain is not imaged.

After a head bar of choice is secured with glue and dental acrylic and the skull is covered with glue. Using a fine tip marker, two parallel lines (between lambda and bregma) is drawn until the lateral ridges as outlines for craniotomy. After the drilling is complete, “the typewriter method” is used to remove the bone. A fitting strip of PMP is placed in the craniotomy. The surroundings are then sealed with glue while holding the PMP down with a pair of forceps. The attachment is then further secured with glue and acrylic.

*** Since PMP is a deformable polymer, the exposure needs to be physically protected when the brain is not being imaged. You can use a cap attached to head bar to the craniotomy safe Please see Supplementary Figure 9, right panel).

iv- Reflective Prism Insertion

In some experiments, brain imaging through implanted reflective prism is performed. Some previous studies leveraged the anatomy by implanting the prisms in fissures, so no cortical incisions were necessary (Low RJ *et al.*). Here we describe a transcortical implantation of prisms.

Following general guidelines for craniotomy, after the skin incision, removing the subcutaneous tissue and periosteum, a head bar of choice is secured with glue and dental acrylic and the skull is covered with glue. Using a fine tip marker, glass is outlined on the bone making sure that the markings do not end on the sinuses. After the craniotomy is complete and bleeding control is complete (if needed), dura is removed.

After the removal of the dura, a vitrectomy blade (Please see Supplementary Figure 10, left panel) is secured on the stereotaxic arm and positioned perpendicular to the brain surface. After careful inspection of the cerebral vasculature, a line that is ~1.5 mm long is defined for cortical incision (Please see Supplementary Figure 10, right panel, dashed green line). The line should be placed avoiding big branches of the vessels to minimize bleeding. The tip of the blade is positioned on this defined line and moved back and forward to cut the cortex surface. For every cut, move ~200-250 *μ*m down to cut through the cortex while irrigating the area with sterile saline for bleeding control.

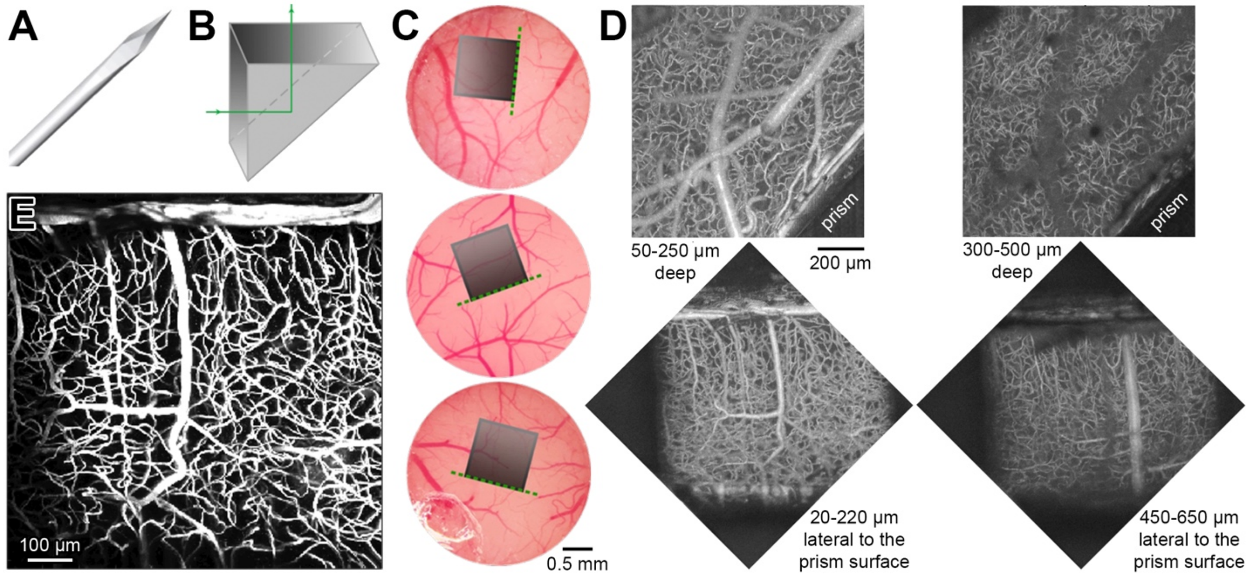

Supplementary Figure 10. Reflective Prism Insertion. A. Tip of a vitrectomy knife. B. Schematic of a prism. C. Examples of incision and prism positioning. Lighter side of the square denotes the tip of the prism. D. OCT angiography images taken next to the prism (top row) and through the prism (bottom row) at different depths. E. MIP of 2-P angiography images taken through the prism with iv FITC-dextran.

A 1 mm reflecting prism (often used ones are aluminum coated on the hypotenuse) is glued to a coverslip with NOA61. A drop of super glue is applied to a sharpened wooden stick to attach to the coverslip keeping the prism on the bottom. Wooden stick is held by the stereotaxic arm and prism edge is lined with the incision (Please see Supplementary Figure 10, right panel, grey box). By slowly lowering the prism/coverslip, the exposure is closed. The coverslip is then sealed with glue and acrylic.

v- Intracerebroventricular (icv) Cannula Insertion

When an excess port is required for the lateral ventricles, icv cannula is commonly used (Please see Supplementary Figure 11, left panel). The cannulas can be implanted chronically. You can implant the cannula in the contralateral hemisphere of the craniotomy (Please see Supplementary Figure 11, right panel).

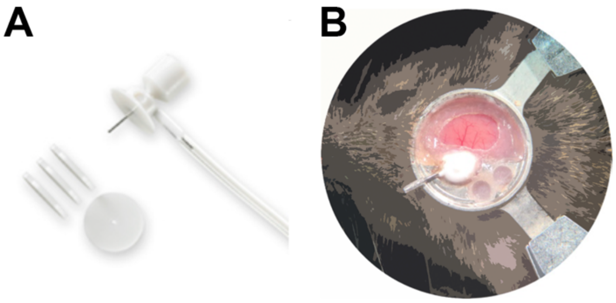

Supplementary Figure 11. Intracerebro-ventricular (icv) Cannula. A. An example of an icv cannula (ALZET Brain Infusion Kit 3). B. icv cannula with half CS craniotomy.

Lateral ventricle coordinates are: 0.1 mm posterior, 1 mm lateral 3 mm deep relative to bregma. These coordinates are marked with a fine tip marker. Using 0.9 mm drill bit, a burr hole is created. Using the arm of the stereotaxic frame, cannula is lowered until the base is securely resting on the skull. It is then secured to the surrounding bone with glue and acrylic. The outer end of the cannula can be connected to an osmotic pump or it may be simply capped to prevent infection. If the latter option is chosen, cap needs to be removed during injections and the end of the cannula needs to be fitted with a tubing. Tubing needs to be filled before attaching to prevent air bubble injection. The other end of the tubing can be attached to a Hamilton syringe or pressure pump. The total volume injected icv should not exceed 10 μl during an injection and the duration of the injection should not be shorter than 10 minutes. After the injection is complete, cannula should be capped again before returning the mouse to their cage.

vi- Making the Imaging "Well"

To assist retaining the liquids during surgery and imaging with immersion objectives, a "well" needs to be created. This well needs to be built with low edges but wide circumference to help for easy surgical access and to accommodate larger objectives.

One common way is to create a well out of fiber washer (In Supplementary Figure 12, left panel, a 3/8" washer is used). Fiber washer is cut with scissors so that two ends would

be in contact with head bar. After gluing the ends to the bar, washer is gently pushed down and inner edge is glued to the skull. Gaps are filled and a thin edge is created using dental acrylic. To achieve a thin and wide layer of acrylic, you can wait 30-60 seconds after application for the acrylic to pre-cure (just cured on the outside but still malleable on the inside) and use a water or saline dipped cotton tip applicator to push the wall of acrylic.

Another way to design and print the well is using a 3-D printer. In this case, the plastic is attached using glue and acrylic (Please see Supplementary Figure 12, right panel).

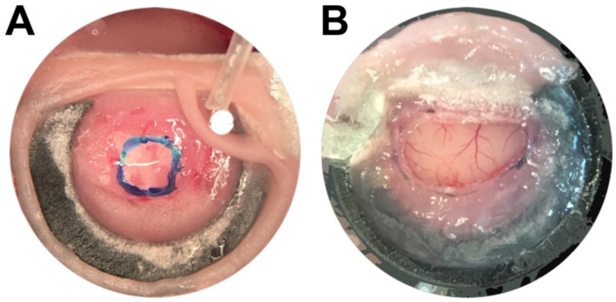

Supplementary Figure 12. Examples of Imaging Well. A. Washer and acrylic imaging well. B. 3-D printed imaging well.

***In the left example, an inner wall is created to protect the screw (ground electrode) from liquid for an electrophysiology experiment.

vii-When Things Go Wrong

Unfortunately, some surgeries could be less than ideal. Regular exposures should be ready to image around day 10-14 after surgery. In some cases, longer healing time may be needed, but if the exposure does not start clearing up after 21 days, the animal should be excluded. Supplementary Figure 13 shows an example of overvascularization after significant inflammation.

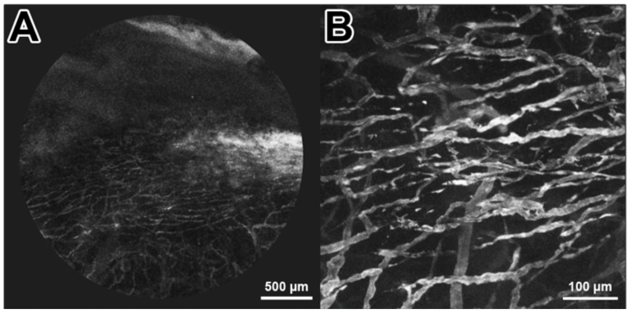

Supplementary Figure 13. Example of an unsuccessful surgery. MIP of 4x (left) and 20x stacks (right) are acquired after iv FITC-dextran injection. Note the dural thickening and over vascularization as well as decreased penetration due to inflammation.

4. SUPPLEMENTARY REFERENCES

- Foley PL, Liang H, Crichlow AR. Evaluation of a sustained-release formulation of buprenorphine for analgesia in rats. J Am Assoc Lab Anim Sci. 2011 Mar;50(2):198-204. PMID: 21439213; PMCID: PMC3061420.
- Green CJ, Knight J, Precious S, Simpkin S. Ketamine alone and combined with diazepam or xylazine in laboratory animals: a 10-year experience. Lab Anim. 1981 Apr;15(2):163-70. doi: 10.1258/002367781780959107. PMID: 7278122.
- Hedley-Whyte ET, Hsu DW. Effect of dexamethasone on blood-brain barrier in the normal mouse. *Ann Neurol*. 1986;19(4):373-377. doi:10.1002/ana.410190411
- Langford DJ, Bailey AL, Chanda ML, et al. Coding of facial expressions of pain in the laboratory mouse. *Nat Methods*. 2010;7(6):447-449. doi:10.1038/nmeth.1455
- Liang P, Shan W, Zuo Z. Perioperative use of cefazolin ameliorates postoperative cognitive dysfunction but induces gut inflammation in mice. J Neuroinflammation. 2018 Aug 22;15(1):235. doi: 10.1186/s12974-018-1274-6. PMID: 30134985; PMCID: PMC6106929.
- Low RJ, Gu Y, Tank DW. Cellular resolution optical access to brain regions in fissures: imaging medial prefrontal cortex and grid cells in entorhinal cortex. Proc Natl Acad Sci U S A. 2014 Dec 30;111(52):18739-44. doi: 10.1073/pnas.1421753111. Epub 2014 Dec 12. PMID: 25503366; PMCID: PMC4284609.
- Schuler B, Rettich A, Vogel J, Gassmann M, Arras M. Optimized surgical techniques and postoperative care improve survival rates and permit accurate telemetric recording in exercising mice. BMC Vet Res. 2009 Aug 2;5:28. doi: 10.1186/1746-6148-5-28. PMID: 19646283; PMCID: PMC2727500.
